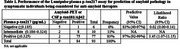# Performance of the Lumipulse plasma *p*‐tau217 assay to assess eligibility for amyloid‐lowering therapies

**DOI:** 10.1002/alz70856_097514

**Published:** 2025-12-24

**Authors:** Alicia Algeciras‐Schimnich, Susan Ashrafzadeh‐Kian, Joshua A Bornhorst, Daniel Figdore, Brian J Burkett, Petrice M Cogswell, Derek R. Johnson, Stuart J McCarter, Jonathan Graff‐Radford, Ronald Petersen, Vijay K. Ramanan

**Affiliations:** ^1^ Mayo Clinic, Rochester, MN, USA; ^2^ Department of Radiology, Mayo Clinic, Rochester, MN, USA; ^3^ Department of Neurology, Mayo Clinic, Rochester, MN, USA

## Abstract

**Background:**

Clinically available plasma *p*‐tau217 assays may be utilized in the determination of eligibility for amyloid‐lowering therapies. Our institution recently introduced a plasma *p*‐tau217 assay in clinical practice using a two‐cutpoint approach that optimizes the accuracy while minimizing false positive risk and categorizing ≤20% of results as intermediate. In this study, we prospectively evaluate the performance of a plasma *p*‐tau217 assay for detection of amyloid pathology using these predefined cutpoints.

**Method:**

Patients recruited were being considered for lecanemab therapy at a tertiary care subspecialty clinic. Plasma *p*‐tau217 was measured using the clinically available Fujirebio Lumipulse *p*‐tau217 assay at Mayo Clinic, and results (pg/mL) were classified as either negative (≤0.185), intermediate (0.186–0.324) or positive (≥0.325). Amyloid pathology was determined by amyloid‐PET and/or CSF *p*‐tau181/Aβ42.

**Result:**

Ninety‐three patients were recruited between November 2023 and December 2024. Of these, 49 underwent amyloid‐PET, 4 had CSF *p*‐tau181/Aβ42 measurements, and 40 had both PET and CSF results (with 3/40 discordant cases), for use in the assessment of amyloid pathology. The three discordant cases were considered amyloid positive for purposes of plasma *p*‐tau217 comparison. Overall amyloid positivity was 90%. Plasma *p*‐tau217 results were positive in 83% (77/93), negative in 6% (6/93), and intermediate in 11% (10/93) of patients. An elevated *p*‐tau217 predicted the presence of amyloid positivity with a diagnostic accuracy of 96% (95% CI 90% − 99%). In the intermediate range, 80% of patients were positive for amyloid pathology. In patients with a definitive *p*‐tau217 result (positive or negative), the positive and negative predictive values were 97% and 83%. There were three patients with discordant results: one PET+/p‐tau217‐, one PET‐/p‐tau217+, and one CSF‐/p‐tau217+. Clinical presentation of these discordant cases was examined, and relevant findings will be discussed.

**Conclusion:**

The Lumipulse plasma *p*‐tau217 assay exhibited acceptable diagnostic accuracy for the determination of amyloid pathology when compared to either amyloid‐PET or CSF *p*‐tau181/Aβ42. This prospective study confirms the utility a two‐cutpoint approach for interpreting plasma *p*‐tau217 for the assessment of amyloid pathology eligibility for amyloid‐lowering therapies in a heterogenous clinical cohort.